# A new species of *Rana* from the Dabie Mountains in eastern China (Anura, Ranidae)

**DOI:** 10.3897/zookeys.724.19383

**Published:** 2017-12-21

**Authors:** Chencheng Wang, Lifu Qian, Chenling Zhang, Weibo Guo, Tao Pan, Jun Wu, Hui Wang, Baowei Zhang

**Affiliations:** 1 Key Laboratory of Eco-engineering and Bio-technique, School of Life Sciences, Anhui University, Hefei, 230601, China; 2 College of life science and chemistry, Jiangsu second normal University, Nanjing, 210013, Jiangsu; 3 Ministry of Environmental Protection, Nanjing Institute of Environmental Sciences, Nanjing 210042, Jiangsu, China

**Keywords:** Amphibians, morphology, molecular phylogeny, taxonomy

## Abstract

A new species *Rana dabieshanensis* **sp. n.** is described from the Dabie Mountains in Anhui Province, China, based on morphological character differences and molecular analyses. The new species can be distinguished from its congeners by a combination of diagnostic characters. The results of phylogenetic analyses (based on 12s rRNA, 16s rRNA, ND2, Cyt *b*, RAG1, BDNF and Tyr) and genetic distances (based on Cyt *b*) indicate that the new species belongs to the *Rana longicrus* group, and is placed as the sister taxon to *R. hanluica*.

## Introduction

The true frogs of the genus *Rana* Linnaeus are broadly distributed across Eurasia and the Americas (Amphibia Web 2017, Frost 2016, Yuan et al. 2016). Because of their body coloration and habitat preferences, the species of this genus are colloquially known as brown frogs or wood frogs (Boulenger 1920, Yan et al. 2011). To date, more than 100 species are attributed to this genus, with their distribution ranges across Asia (32 species), Europe and the Near East (12 species), and the Americas (57 species) with several lineages still being not formally described (Yuan et al. 2016, Amphibia Web 2017). In China, the genus *Rana* contained 17 species (Fei et al. 2012) that were divided into three groups (*R. longicrus* group, *R. chensinensis* group, and *R. amurensis* group) based on morphology and distribution (Fei et al. 2009). Molecular phylogenies, however, indicate presence of *R. longicrus*, *R. amurensis*, *R. chensinensis*, *R. sauteri*, *R. johnsi*, *R. shuchinae*, and *R. weiningensis* groups within the Chinese *Rana* sensu lato (Che et al. 2007, Yuan et al. 2016). And 24 species are contains in Chinese *Rana* genus (AmphibiaChina 2017, Yuan et al. 2016, Zhao et al. 2017). The species of the *R. longicrus* group are widely distributed in southern and eastern China, and the recent surge in new species descriptions suggests that these still insufficiently explored regions may contain many undescribed cryptic species (Lu and Li 2001, Lu et al. 2007, Shen et al. 2007, Li et al. 2008, Yan et al. 2011).

From 2015 to 2016, we collected 17 specimens of *Rana* sp. in montane forests of the Dabie Mountains. The specimens exhibited comparatively large body size and a straight dorsolateral fold from posterior corner of eye to groin. Initially, they were identified as *R. omeimontis* (according to the identification key by Ye et al. 1993). However, from the further detailed studies indicated that this record might represent a yet undescribed cryptic species of *Rana*.

Generally, the brown frogs are difficult to identify in the field because of their close morphological similarities especially when closely related species have overlapping distributions (Stuart et al. 2006, Che et al. 2007). According to the results of subsequent molecular analyses and morphological identification, we confirmed that the specimens of *Rana* sp. from Dabie Mountains were distinct from any species presently recognized in the genus of *Rana* and herein we describe them as a new species.

## Materials and methods

**Figure 1.**
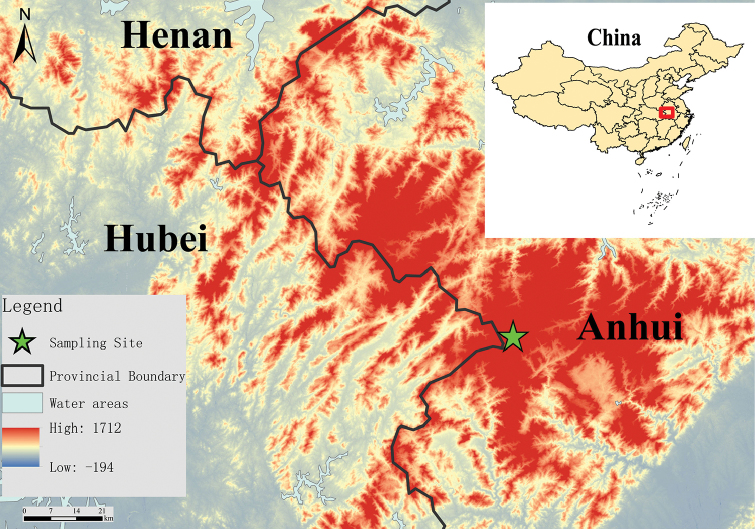
Distribution of *Rana dabieshanensis* sp. n. in Dabie Mountains (Anhui, Hubei, and Henan provinces, central China). Occurrence record is marked with green mark.

**Sampling**: In total, 17 specimens of *Rana* sp. were collected from Yaoluoping National Nature Reserve in Dabie Mountains, Yuexi County, Anhui Province, China (30°58'16.92"N, 116°04'11.88"E, elevation 1150 m a.s.l.) (Fig. 1), in August 2015 and August 2016. Those individuals were dipped in 10% formalin (10 seconds) for fixation and subsequently transferred into 75 % ethanol for storage. Before fixing in formalin, liver tissues from all individuals were sampled and preserved in 100% ethanol for molecular analyses. All specimens and tissue samples were deposited in the Anhui University Museum, Research Center for Biology.

**DNA extraction, PCR amplification and sequencing**: Genomic DNA was extracted from liver tissues of seven *Rana* sp. specimens using the standard proteinase K/phenol-chloroform protocol (Sambrook et al. 1989). Four mitochondrial genes (12S rRNA, 16S rRNA, ND2, and Cyt *b*) and three nuclear DNA markers (Tyr, BDNF and RAG1) were sequenced for one individual, while for six remaining specimens only Cyt *b* mtDNA gene was sequenced. The primers used for PCR and sequencing are summarized in Table 1. All PCRs were performed with the same conditions in 50 μL: 20 to 80ng of genomic DNA, 25 μL 2×EasyTaq PCR SuperMix polymerase (TransGen Biotech, containing 1.25U Ex Taq, 0.4mM dNTP, 4mM Mg^2+^) and 0.4 μM of primers. Reactions were performed with the following profile: PCR cycles were 5 min at 95 °C followed by 35 cycles of 30 s at 95 °C, 30 s at appropriate annealing temperature (Table 1), and 1 min at 72 °C, with a final extension at 72 °C for 10 min. The PCR products were purified using an EasyPure PCR Purification Kit (TransGene), and sequenced directly using the primers used in PCRs and the BigDye Terminator v3.0 Ready Reaction Cycle Sequencing Kit (Applied Biosystems) following the manufacturer’s instructions on an ABI Prism 3730 automated sequencer.

**Table 1.** Primers used for PCR and sequencing.

Locus	Primer Name	Sequences (5’ end 3’ end)	Temperature (°C)	Source
12S	L2519	AAACTGGGATTAGATACCCCACTAT	50	Kocher et al. (1989)
	H3296	GCTAGACCATKATGCAAAAGGTA	50	Kocher et al. (1989)
16S	16SAR	AACGCTAAGATGAACCCTAAAAAGTTCT	50	Kocher et al. (1989)
	R16	ATAGTGGGGTATCTAATCCCAGTTTGTTTT	50	et al. (2000)
ND2	HERP322	TYCGARGACAGAGGTTTRAG	42	Yuan et al. (2016)
	HERP323	CAYCCACGRGCYATYGAA	42	Yuan et al. (2016)
Cyt *b*	HERP328	GAAAARCTRTCGTTGTWATTCAACTA	50	Yuan et al. (2016)
	HERP329	CTACKGGTTGTCCYCCRATTCATGT	50	Yuan et al. (2016)
Tyr	Tyr1G	TGCTGGGCRTCTCTCCARTCCCA	50	Bossuyt and Milinkovitch (2000)
	Tyr1B	AGGTCCTCYTRAGGAAGGAATG	50	Bossuyt and Milinkovitch (2000)
RAG1	AmpF2	ACNGGNMGICARATCTTYCARCC	52	Hoegg et al. (2004)
	AmpR2	GGTGYTTYAACACATCTTCCATYTCRTA	52	Hoegg et al. (2004)
BDNF	BDNF 2F	GAGTGGGTCAAGAGGAGG	41	Zhou et al. (2012)
	BDNF_2R	ACTGGGTAGTTCGGCATT	41	Zhou et al. (2012)

**Phylogenetic analyses**: 147 sequences were used for genetic analysis, which include 135 sequences within the Chinese *Rana* were downloaded from NCBI and 12 sequences in this study. The data are summarized in Table 2. All nucleotide sequences were aligned using MUSCLE (Edgar 2004) with default parameters and checked manually with MEGA 5.0 (Tamura et al. 2011), the length of the fragments was trimmed; newly obtained sequences were deposited in GenBank (Table 2). Nucleotide sites with ambiguous alignments were deleted from the analyses. Bayesian inference (BI) and maximum likelihood (ML) analyses were conducted using the six concatenated gene fragments. BI analyses were performed in MRBAYES v3.1.2 (Ronquist and Huelsenbeck 2003) using the optimal partitioning strategy and best-fit nucleotide substitution model for each region (Table 3) selected by PARTITIONFINDER v1.1.1 (Lanfear et al. 2012). MRBAYES analyses simultaneously initiated two Markov Chain Monte Carlo (MCMC) model runs to provide additional confirmation of convergence of posterior probability distributions. Analyses were run for 10,000,000 generations. Chains were sampled every 1000 generations. The first 25% of the total trees were discarded as "burn-in" and the remaining trees were used to generate a majority-rule consensus tree and to calculate Bayesian posterior probabilities. Nodal support was further assessed with a maximum-likelihood (ML) analysis in RAXML V.7.0.3 with 1000 bootstraps. *Pelophylax nigromaculatus* sequences were downloaded from GenBank and used as outgroup.

**Table 2.** Species, sample localities, voucher museum numbers and GenBank accession numbers for DNA sequences of *Rana* species used in the phylogenetic analyses.

Species	Locality	Voucher	GenBank No.						Reference
			12S-16S	Cyt *b*	ND2	RAG1	BDNF	Tyr	
*R. dabieshanensis* sp. n. (1)	China: Anhui Province: Dabie Mountains area	AHU2016R001	MF172963	MF172964	MF172974	MF172971	MF172972	MF172973	This Study
*R. dabieshanensis* sp. n. (2)	China: Anhui Province: Dabie Mountains area	AHU2016R002	N/A	MF172965	N/A	N/A	N/A	N/A	This Study
*R. dabieshanensis* sp. n. (3)	China: Anhui Province: Dabie Mountains area	AHU2016R003	N/A	MF172966	N/A	N/A	N/A	N/A	This Study
*R. dabieshanensis* sp. n. (4)	China: Anhui Province: Dabie Mountains area	AHU2016R004	N/A	MF172967	N/A	N/A	N/A	N/A	This Study
*R. dabieshanensis* sp. n. (5)	China: Anhui Province: Dabie Mountains area	AHU2016R005	N/A	MF172968	N/A	N/A	N/A	N/A	This Study
*R. dabieshanensis* sp. n. (6)	China: Anhui Province: Dabie Mountains area	AHU2016R006	N/A	MF172969	N/A	N/A	N/A	N/A	This Study
*R. dabieshanensis* sp. n. (7)	China: Anhui Province: Dabie Mountains area	AHU2016R007	N/A	MF172970	N/A	N/A	N/A	N/A	This Study
*R. amurensis*	Russia: Tomskaya: Teguldetskii district	MSUZP-SLK-RUS49	KX269203	KX269349	KX269418	KX269568	KX269278	KX269795	Yuan et al. 2016
*R. arvalis*	Russia: Mordovia: Chamzinskii district	MSUZP-SLK-MKR21	KX269197	KX269344	KX269413	KX269562	KX269272	KX269789	Yuan et al. 2016
*R. asiatica*	China: Xinjiang: 47tuan	KIZ-XJ0251	KX021945	KX021945	KX021945	KX269565	KX269275	KX269792	Yuan et al. 2016
*R. chaochiaoensis*	China: Sichuan: Zhaojue	SCUM0405170CJ	KX269192	KX269339	KX269408	KX269557	KX269267	KX269800	Yuan et al. 2016
*R. chensinensis*	China: Shaanxi: Huxian	KIZ-RD05SHX01	KX269186	KX269333	KX269402	KX269551	KX269261	KX269779	Yuan et al. 2016
*R. culaiensis*	China: Shandong: Culaishan shan	KIZ-SD080501	KX021986	KX021986	KX021986	KX269555	KX269265	KX269783	Yuan et al. 2016
*R. dybowskii*	Russia: Primorye region: Khasanskii District	MSUZP-IVM-1d	KX021949	KX021949	KX021949	KX269553	KX269263	KX269781	Yuan et al. 2016
*R. hanluica*	China: Guangxi: Maoershan shan	KIZGX07112915	KX269191	KX269338	KX269407	KX269556	KX269266	KX269784	Yuan et al. 2016
*R. huanrenensis*	South Korea	MMS 231	KX021944	KX021944	KX021944	KX269548	N/A	KX269776	Yuan et al. 2016
*R. japonica*	Japan: Isumi-shi: Chiba Prefecture	KIZ-YPX11775	KX269220	KX269364	KX269434	KX269585	KX269295	KX269811	Yuan et al. 2016
*R. jiemuxiensis*	China: Hunan: Jiemuxi	KIZ-HUN0708013	KX269221	KX269365	N/A	KX269586	KX269296	KX269812	Yuan et al. 2016
*R. kukunoris*	China: Qinghai: Qinghai Lake	KIZCJ06102001	KX021947	KX021947	KX021947	KX269550	KX269260	KX269778	Yuan et al. 2016
*R. kunyuensis*	China: Shandong: Kunyu shan	KIZ-HUI040001	KX269201	KX269347	KX269416	KX269566	KX269276	KX269793	Yuan et al. 2016
*R. longicrus*	China: Taiwan: Xiangtianhu: Miaosu	NMNS15022	KX269189	KX269336	KX269405	KX269554	KX269264	KX269782	Yuan et al. 2016
*R. omeimontis*	China: Sichuan: Zhangcun: Hongya	SCUM0405196CJ	KX021946	KX021946	KX021946	KX269558	KX269268	KX269785	Yuan et al. 2016
*R. zhenhaiensis*	China: Zhejiang: Zhenhai	KIZ0803271	KX269218	N/A	KX269433	KX269583	KX269293	KX269809	Yuan et al. 2016
*R. coreana*	South Korea	MMS 223	KX269202	KX269348	KX269417	KX269567	KX269277	KX269794	Yuan et al. 2016
*R. sauteri*	China: Taiwan: Kaohsiung	SCUM0405175CJ	KX269204	KX269350	KX269419	KX269569	KX269796	KX269279	Yuan et al. 2016
*R. zhengi*	China: Sichuan: Hongya: Zhangcun	SCUM0405190CJ	KX269206	KX269352	KX269421	KX269571	KX269798	KX269495	Yuan et al. 2016
*R. johnsi*	Vietnam: Lam Dong: Loc Bao	ABV 00203	KX269182	KX269328	KX269398	KX269546	KX269774	KX269471	Yuan et al. 2016
*R. shuchinae*	China: Sichuan: Zhaojue	CIB-HUI040009	KX269210	KX269356	KX269425	KX269575	DQ360057	KX269499	Yuan et al. 2016
*R. weiningensis*	China: Sichuan: Weining	SCUM0405171	KX269217	KX269362	KX269432	KX269582	KX269808	KX269506	Yuan et al. 2016
*Pelophylax nigromaculatus*	China: Sichuan: Hongya	SCUM-045199CJ	KX269216	KX269361	KX269431	KX269581	KX269807	KX269505	Yuan et al. 2016

**Table 3.** Sequence information (a) and results of model selection by PartitionFinder (b). “V” and “PI” indicated the variable sites and parsimony-informative sites of each locus, respectively.

(a)			
**Sequence name**	**Sequence length (bp)**	**V**	**PI**
12-16s rRNA	1743	555	336
Cyt *b*	834	306	262
ND2	726	392	323
RAG1	1191	158	86
Tyr	456	73	31
BDNF	454	31	10
(b)			
**Best fit model**	**Partitions**		
GTR+I+G	12S-16S, ND2-2nd		
HKY+I	RAG-3rd, Tyr-1st, Tyr-3rd, BDNF-1st, BDNF-2nd		
SYM+I+G	Cyt *b*-1st		
HKY+I+G	ND2-3rd, RAG1-1st, RAG1-2nd, Cyt *b*-2nd, Tyr-2nd		
GTR+I	ND2-1st, Cyt *b*-3rd		
K80+I	BDNF-3rd		

Apart from phylogenetic tree-based methods, we also calculated pairwise sequence divergence based on uncorrected *p*-distance using MEGA 5.0 (Tamura et al. 2011) to determine the genetic distance between species. The analysis compared the 7 individuals of *Rana* sp. from the Dabie Mountains to other 22 species of the genus *Rana* inhabiting China.

**Morphological analyses**: The morphometric data were examined for 10 individuals. Measurements were made by Yanan Zhang using a vernier caliper with a precision of 0.1 mm. 17 linear measurements (Fei et al. 1999) were taken as follows:

**SVL** (snout-vent length, from tip of snout to vent);

**HL** (head length, from posterior corner of mandible to tip of snout);

**HW** (head width, the greatest cranial width);

**SL** (snout length, from tip of snout to the anterior corner of the eye);

**IN** (internarial distance);

**ED** (horizontal eye diameter);

**IO** (interorbital distance, the minimal distance between upper eyelids);

**UE** (upper eyelid width, the maximal width of upper eyelid);

**TD** (horizontal tympanic diameter);

**LAHL** (length of lower arm and hand, from the tip of finger III to the elbow joint);

**HAL** (hand length, from proximal end of outer palmar tubercle to tip of the third finger);

**LAD** (diameter of lower arm);

**HLL** (hind limb length, from the tip of the toe IV to groin);

**TL** (tibia length);

**TW** (tibia width, the greatest width of tibia);

**FL** (foot length, from the proximal end of the inner metatarsal tubercle to the tip of the toe IV) and

**TFL** (length of tarsus and foot, from the proximal end of tarsus to the tip of the fourth toe IV).

The description of toe webbing followed Savage (1975) (Table 4). The morphological characters of the individuals of *Rana* sp. from Dabie Mountains were compared with the members in the *R. longicrus* group, *R. chaochiaoensis* Liu, 1946, *R. culaiensis* Li, 2008, *R. hanluica* Shen, Jiang & Yang 2007, *R. jiemuxiensis* Yan, 2011, *R. longicrus* Stejneger, 1898, *R. maoershanensis* Lu, 2007, *R. omeimontis* and *R. zhenhaiensis* Ye, 1995. We also compared individuals collected in this area with other *Rana* species distributed in other parts of China *R. arvalis* Gislén, 1959 (previously listed as *R. altaica*, identification following Yang et al. 2010), *R. amurensis* Boulenger, 1886, *R. luanchuanensis* Zhao, 2017, *R. asiatica* Bedriaga, 1898, *R. chensinensis* David, 1875, *R. dybowskii* Guenther, 1876, *R. huanrenensis* Liu, 1993, *R. kukunoris* Nikolsky, 1918 and *R. kunyuensis* Lu, 2002. Considering the restrictions of samples, the morphological characteristics of the species in *Rana* were obtained from literature (Fei et al. 2009, Fei et al. 2010, Fei et al. 2012, Zhao et al. 2017, Li et al. 2008, Yan et al. 2011, Inger et al. 1989).

**Table 4.** Measurements [in mm; mean±SD (range)] of adult specimens of *Rana dabieshanensis* sp. n.

Character	*R. dabieshanensis* sp. n				
	Holotype	Males (8)	Mean±SD	Females (2)	Mean
SVL	62.8	50.9–62.8	57.1±4.1	53.0–68.3	60.7
HL	17.8	16.0–19.0	17.8±1.1	18.0–19.7	18.8
HW	17.6	15.3–18.9	17.4±1.1	16.7–18.8	17.8
SL	8.4	7.5–9.5	8.4±0.8	7.9–8.5	8.2
IN	5.5	3.9–5.5	4.5±0.6	4.3–4.6	4.5
IO	5.5	3.9–5.5	4.5±0.6	4.3–4.5	4.4
UE	3.6	2.9–3.8	3.4±0.4	3.6–3.7	3.6
ED	4.8	4.1–5.7	4.8±0.6	4.6–4.7	4.5
TD	4.0	3.5–5.2	4.4±0.6	4.2–4.3	4.3
LAHL	27.6	21.4–27.6	24.7±2.1	23.4–26.7	24.5
LAD	8.1	5.6–8.1	6.6±1.2	4.9–8.1	5.2
HAL	14.8	13.1–14.8	13.8±0.7	13.3–13.6	13.5
HLL	129.1	100.4–129.1	115.5±10.6	102.4–121.4	111.9
TL	40.6	31.4–40.6	35.1±3.3	30.4–37.6	34.0
TW	8.4	5.9–8.4	7.5±0.9	6.4–6.7	6.5
TFL	53.2	43.1–53.2	48.6±3.4	44.3–51.2	47.7
FL	35.5	27.6–35.1	32.8±2.8	27.8–35.6	31.7

## Results

**Figure 2.**
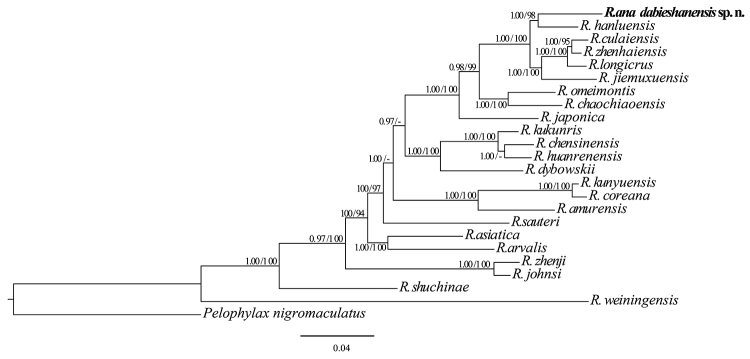
The Bayesian consensus tree resulting from analysis of four mitochondrial genes (12S rRNA, 16S rRNA, ND2 and Cyt *b* genes) and three nuclear genes (Tyr, RAG1 and BDNF) dataset for Chinese *Rana* species. The new species is in bold. Number near the nodes are Bayesian posterior probabilities / Maximum Likelihood bootstrap values but only when values are ≥ 0.95 and ≥ 70, respectively.

**Molecular phylogenetic analyses**: The BI and ML phylogenetic tree were constructed based on concatenated DNA sequences of the mitochondrial genes and nuclear genes (12S rRNA, 16S rRNA, ND2, Cyt *b*, Tyr, BDNF, and RAG1) with a total length of the final alignment 5414 bp. Besides, the variable sites and potentially parsimony informative sites are listed in Table 3. The results by BI and ML displayed the same topologies and strong node supporting values (Fig. 2). The major clades were similar to previous studies (Yuan et al. 2016). The individuals of *Rana* sp. from Dabie Mountains, clustered in the *R. longicrus* group and are reconstructed as a sister species of *R. hanluica* with high node support values (1.0/100 for BI posterior probabilities / ML bootstrap, respectively) (Fig. 2).

Furthermore, the nucleotide sequence divergences based on uncorrected pairwise distances model among the 23 brown frog species examined are shown in Table 5. The sequence divergences among the new populations from Dabie Mountains were 0.2%. Sequence divergences between the new populations from Dabie Mountains congeners ranged from 8.6% (*R. culaiensis*) to 27.7% (*R. weiningensis*). Within the *R. longicrus* group, divergence between were from 8.6% (*R. culaiensis*) to 16.0% (*R. chaochiaoensis*). Sequence divergence between the individuals of *Rana* sp. from Dabie Mountains and the sister species *R. hanluica* is 8.8% (Table 5).

Based on phylogenetic analysis of both nuDNA and mtDNA genetic markers and genetic distances in Cyt *b* mtDNA gene, it is demonstrated that the population of *Rana* sp. from the Dabie Mountains represents a phylogenetically independent evolutionary lineage, and a member of *R. longicrus* group. It represents a previously undescribed species which is described herein.

**Table 5.** The pairwise uncorrected *p*-distance (%) of the Cyt *b* partial sequence (834 bp) used in this study. 1: *Rana dabieshanensis* sp. n; 2: *R. culaiensis*; 3: *Rana longicrus*; 4:*R. zhenhaiensis*; 5:*R. chaochiaoensis*; 6: *R. hanluica*; 7: *R. huanrenensis*; 8: *R. japonica*; 9: *R. jiemuxiensis*; 10:*R. omeimontis*; 11:*R. chensinensis*; 12: *R. dybowskii*; 13: *R. kukunoris*; 14: *R. amurensis*; 15: *R. arvalis*; 16: *R. asiatica*; 17: *R. coreana*; 18: *R. johnsi*; 19: *R. kunyuensis*; 20 : *R. sauteri*; 21: *R. shuchinae*; 22: *R. weiningensis*; 23: *R. zhengi*. The number in bold present the distance between *Rana dabieshanensis* sp. n. and the species of *Rana* analyzed in this study.

	1	2	3	4	5	6	7	8	9	10	11	12	13	14	15	16	17	18	19	20	21	22	23
**1**	**0.002**																						
**2**	**0.086**																						
**3**	**0.101**	0.042																					
**4**	**0.100**	0.032	0.053																				
**5**	**0.160**	0.148	0.159	0.141																			
**6**	**0.088**	0.086	0.086	0.091	0.133																		
**7**	**0.172**	0.197	0.208	0.190	0.171	0.186																	
**8**	**0.148**	0.139	0.161	0.141	0.144	0.138	0.172																
**9**	**0.104**	0.109	0.112	0.116	0.156	0.093	0.193	0.145															
**10**	**0.093**	0.090	0.102	0.106	0.167	0.085	0.193	0.150	0.111														
**11**	**0.178**	0.178	0.190	0.176	0.156	0.180	0.063	0.160	0.186	0.191													
**12**	**0.164**	0.173	0.178	0.169	0.183	0.181	0.121	0.162	0.174	0.188	0.132												
**13**	**0.172**	0.173	0.181	0.171	0.156	0.175	0.059	0.151	0.178	0.182	0.066	0.112											
**14**	**0.184**	0.207	0.219	0.213	0.194	0.186	0.198	0.196	0.193	0.172	0.182	0.191	0.187										
**15**	**0.206**	0.206	0.226	0.213	0.201	0.204	0.174	0.166	0.214	0.211	0.186	0.177	0.168	0.188									
**16**	**0.160**	0.157	0.172	0.166	0.184	0.162	0.163	0.148	0.172	0.166	0.152	0.167	0.146	0.155	0.136								
**17**	**0.199**	0.197	0.206	0.195	0.194	0.190	0.209	0.204	0.191	0.196	0.191	0.189	0.189	0.144	0.199	0.180							
**18**	**0.185**	0.183	0.187	0.177	0.196	0.194	0.208	0.181	0.184	0.192	0.189	0.186	0.187	0.210	0.192	0.191	0.219						
**19**	**0.201**	0.199	0.208	0.200	0.199	0.197	0.212	0.199	0.202	0.193	0.197	0.196	0.190	0.152	0.200	0.185	0.022	0.220					
**20**	**0.193**	0.192	0.209	0.193	0.192	0.181	0.197	0.169	0.204	0.201	0.183	0.182	0.177	0.176	0.192	0.155	0.199	0.222	0.192				
**21**	**0.191**	0.207	0.235	0.210	0.220	0.210	0.193	0.205	0.211	0.197	0.198	0.189	0.182	0.202	0.194	0.182	0.206	0.197	0.194	0.204			
**22**	**0.277**	0.279	0.262	0.266	0.255	0.267	0.271	0.252	0.283	0.267	0.259	0.267	0.254	0.261	0.277	0.237	0.273	0.292	0.270	0.273	0.259		
**23**	**0.203**	0.195	0.202	0.185	0.192	0.201	0.198	0.194	0.193	0.211	0.191	0.203	0.185	0.217	0.192	0.194	0.226	0.046	0.222	0.229	0.204	0.282	

### Taxon description

#### Rana dabieshanensis sp. n.

Taxon classificationAnimaliaAnuraRanidae

http://zoobank.org/B2595A92-AD54-433B-9F0A-C05DF5E68336

##### Holotype.

**Figure 3.**
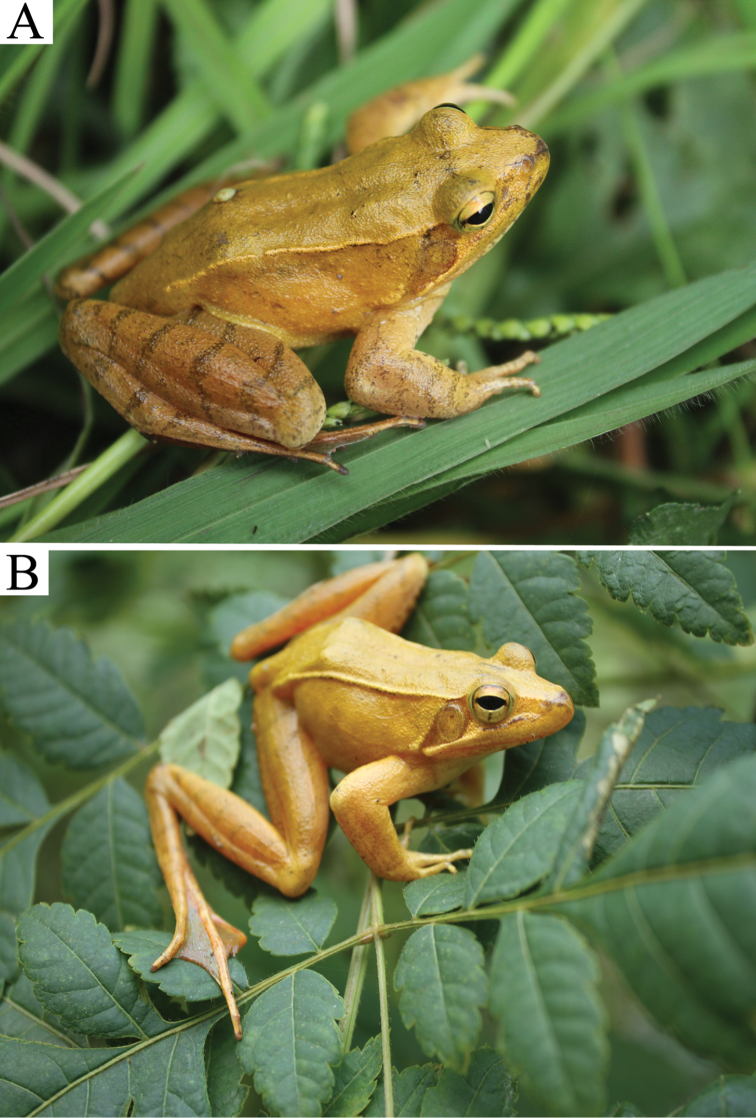
Dorsal (**A**) and lateral view (**B**) of the holotype (AHU2016R001, male) of *Rana dabieshanensis* sp. n. in life.

**Figure 4.**
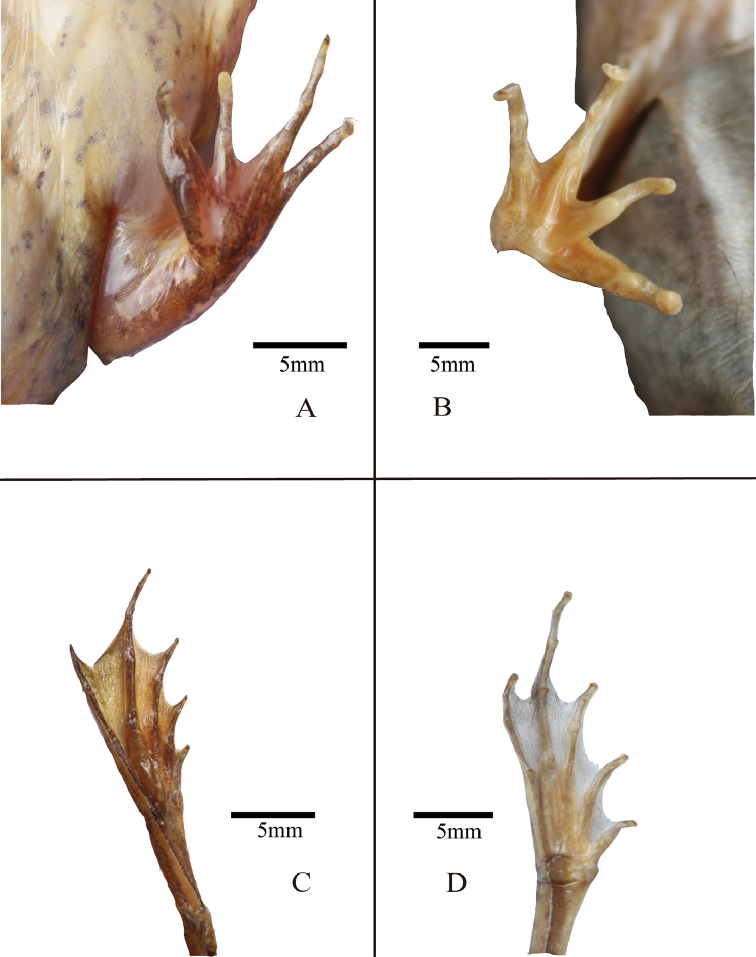
**A** volar view of the left hand of the holotype in life (AHU2016R001, male) **B** volar view of the right hand of female paratype in preservative (AHU2016R010, famle) **C** thenar view of the right foot of the holotype in life (AHU2016R001, male) **D** thenar view of the right foot of the female paratype in preservative (AHU2016R010, famle).

Specimen AHU2016R001, an adult male (Figures 3, 4) from the Yaoluoping National Nature Reserve, Yuexi County, Anhui Province, China (30°58'16.92"N, 116°04'11.88"E, elevation 1150 m a.s.l.) (Fig. 1), leg. Lifu Qian, 8 August, 2016.

##### Paratypes.

Seven males: AHU2016R002, AHU2016R003, AHU2016R004, AHU2016R005, AHU2016R006, AHU2016R007 and AHU2016R008, collected from the same locality as the holotype by Chencheng Wang between 15 and 20 August 2015. Two adult females, AHU2016R009 and AHU2016R010 collected by Lifu Qian at the same locality and time as the holotype.

##### Diagnosis.

The new species is assigned to the genus *Rana* based on the morphological characteristics typical for this genus, including the possession of a prominent dorsolateral folds, dark temporal mask, and a body that is counter-shaded in various shades of brown. The species can be distinguished from its congeners by the following combination of morphological characteristics: (1) comparatively large body size (SVL 50.9–62.8 mm in males, N = 8 and females 53.0–68.3 mm, N = 2); (2) snout obtusely pointed in lateral view; (3) temporal fold distinct; (4) canthus rostralis distinct; (5) dark mask covering tympanum; (6) tympanum diameter equal to eye diameter (7) head length almost equal with head width (8) distinct transverse grayish brown bars on dorsal surface of lower arms, tarsus, thighs, and tibia; (9) dorsal skin smooth, small granules on legs, large tubercles absent; (10) tips of fingers not expanded, relative finger lengths III > I > IV > II, fingers webbing absent, toes two third webbed, toes webbing formula I 2–1– II 2^+^– 1^+^ III 3–2 IV 2–2^+^ V; (11) gray-blackish nuptial pad prominent and forming two groups in males, with minute nuptial spines; (12) external vocal sac absent; (13) a straight dorsolateral fold from temporal area to groin. (14) dorsum coloration varies from golden to brown.

##### Description of Holotype.

SVL 62.8 mm. Head length is approximately equal to the head width (HL/HW = 1.01); snout long and rounded in profile, projecting a little beyond the lower jaw; internarial space equal to the interorbital space (INS/IOS = 1); diameter of the eye larger than the width of upper eyelid (ED/UE=1.33); canthus rostralis distinct; tympanum rounded, with the obvious tympanic rim; tongue deeply notched behind; external vocal sacs not discernable; pupil horizontal.

*Forelimbs*: forearm robust, fingers slender, finger webbing absent; fingertips obtuse with no expansion and lacking circummarginal grooves; relative finger lengths III > I > IV > II; one prominent subarticular tubercle on fingers I and II, two small subarticular tubercles on fingers III and IV; the inner metatarsal tubercle oval-shaped and elongated; outer metatarsal tubercle small rounded; the nuptial pad appeared on the finger I, covered by small black spines and divided into two groups, one near tip lager than the other one.

*Hindlimbs*: hind limbs long (HLL 129.1 mm, 205.7% of SVL), about 4.7 times than length of forelimbs (LAHL 27.6 mm, 43.9% of SVL); heels overlapping when limbs are held at right angles to body; the tibio-tarsal joint reaches beyond the snout-tip when the hind limb is stretched forward; the relative toe lengths IV > III > V > II > I; toes two third webbed, toes webbing formula: I 2 – 1– II 2^+^– 1^+^ III 3 – 2 IV 2 – 2^+^ V and the webbing of the toe IV reaches as far as the penultimate distal joint; toe tips rounded, lacking circummarginal grooves; three tubercles on the IV toes, two tubercles on II III and V toes, one tubercle on I toe; the inner metatarsal tubercle ovoid, small but distinct; without outer metatarsal tubercles.

*Skin*: skin on dorsum is smooth while some small tubercles present on the body flanks and mouth angle; a mass of small tubercles on the dorsal surfaces of thighs and shanks while little warts on forelimb basis; a triangular gray patch behind the eye and anterior to the temporal fold; temporal fold distinct, extending from posterior margin of eye above and behind tympanum to above arm insertion; dorsolateral fold obvious and straight from the temporal area to groin; the throat, chest, belly and ventral surfaces of thighs being smooth with irregular black spots.

*Coloration*: in life and in preservative: in life, the iris is golden with a black pupil, two dark spots near pupil edges in the anterior and posterior edges of eye and a dark vertical bar in the lower half of iris; the color of the dorsal side changes according to environment, from golden to light brown; lip is golden brown with darker brown markings lasting from the area under the eye towards nostrils and snout tip; the mandible whitish with unclear gray spots; large triangular brown patch behind the eye and anterior to temporal fold; forelimbs dorsally the same color as the dorsal surface of body, with four faint ash black stripe in the forearm; the dorsum of the thigh and tibia is a grayish brown, with nine ash black stripes; the sides of the tarsus and foot are grayish brown with three ash black bars; throat, chest, and belly white with irregular black spots; nuptial pad grayish brown. In preservative, dorsal surface gray-brown; all ash black fade to black; throat, chest, and abdomens fade to creamy white, with gray spots.

##### Variation and sexual dimorphism.

Morphometric data were summarized in Table 4. Body size of males (SVL 50.1–62.8 mm) is smaller than that of females (SVL: 53.0–68.3 mm). Their dorsal color varied from golden to dark brown. Number of grayish brown crossbars on dorsal surface of lower arms, tarsus, thighs, and tibia varied. Nuptial pads are absent only in females.

##### Measurements (in mm) of the holotype.

SVL: 62.8; HW: 17.6; HL: 17.8; SL: 8.4; IN: 5.5; IO: 5.5; UE: 3.6; ED: 4.8; TD: 4.0; LAHL: 27.6; LAD: 8.1; HAL: 14.8; HLL: 129.1; TL: 40.6; TW: 8.4; TFL: 53.2; FL: 35.5.

##### Etymology.

The epithet of the new species “dabieshanensis” is a Latinized toponymic adjective derived from the Dabie Mountains in central China where the new species was discovered.

##### Common names.

We recommend the “Dabie Mountain Brown Frog” as a common name of the new species in English; “Da Bie Shan Lin Wa” in Chinese.

##### Ecological notes.

*Rana dabieshanensis* sp. n. appears closely associated with high altitudes of the southeastern mountains environments. Specimens were found at night between 20:00 and 01:00 h around a water pool in Yaoluoping National Nature Reserve, Yuexi, Anhui province, China (Figure. 1). The surrounding habitat consists of small hardwoods, mixed with shrubs and vines. Most of the specimens were found in grass nearby the water, few frogs were in the water. Air temperature was about 13.6 to 17.1 °C and water temperature about 12.1 °C to 14.7 °C. The relative humidity in this area was from 62 to 81%. Other amphibian species include *R. chensinensis*, *Rhacophorus anhuiensis*, *Pelophylax nigromaculata*, *Fejervarya multistriata*, and *Yerana yei* was also recorded during field survey in Yaoluoping National Nature Reserve (Pan et al. 2014).

**Figure 5.**
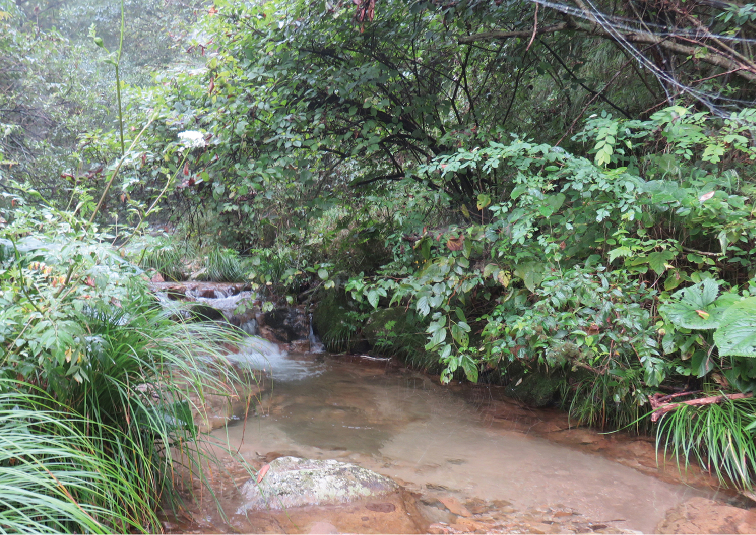
Typical habitat of *Rana dabieshanensis* sp. n. in Dabie Mountains, Anhui Province, China.

##### Distribution.

Currently, *Rana dabieshanensis* sp. n. is only found in the Yaoluoping National Nature Reserve (Anhui Province). This species might be found in other regions of the Dabie Mountains.

##### Comparisons.

*Rana dabieshanensis* sp. n. differ from the Chinese species of the genus *Rana* by following morphological characters: (1) without black glandular ridge in scapular region (vs. an inverted V-shaped black glandular ridge in scapular region in *R. chaochiaoensis*, *R. hanluica*, *R. longicrus*, *R. omeimontis*, *R. maoershanensis*, *R. huanrenensis*, *R. japonica*, and *R. jiemuxiensis*); (2) smooth dorsum without tubercles (vs. many tubercles on the dorsolateral surface in *R. arvalis*, *R. amurensis*, *R. asiatica*, *R. dybowskii*, *R. japonica* and *R. kukunoris*) ; (3) tympanum diameter equal to eye diameter (TD 3.5–5.2 mm, ED 4.1–5.7 mm N=8) (vs. tympanum diameter being 2/3 times of eye diameter in *R. chaochiaoensis* (TD 3.0–5.5 mm, ED 4.3–6.3 mm N = 22), *R. hanluica* (TD 3.5–4.8 mm, ED 5.2–7.8 mm N = 16), *R. omeimontis* (TD 4.0–5.5 mm, ED 5.4–6.9 mm N = 20), *R. zhenhaiensis* (TD 2.5–4.0 mm, ED 5.0–6.4 mm N = 25) and *R. culaiensis* (TD 3.4–4.3 mm, ED 5.1–6.7 mm N = 5), *R. japonica* (described by Stejneger and Matsui in 1907); tympanum diameter being 1/2 times of eye diameter in *R. maoershanensis* (TD 3.4–3.8 mm, ED 6.1–6.7 mm N = 3), *R. huanrenensis* (TD 1.9–3.0 mm, ED 4.0–7.0 mm N = 15), *R. kunyuensis* and *R. chensinensis* (TD 2.5–3.0 mm, ED 5.3–6.0 mm N = 8); tympanum diameter being 3/4 times of the eye diameter in *R. jiemuxiensis* (TD 2.5–4.1 mm, ED 2.8–4.2 mm)); (4) internarial distances almost equal to interorbital distances (IOS 3.9–5.5 mm, INS 3.9–5.5 mm N = 8) (vs. interorbital distances larger than internarial distances of in *R. chaochiaoensis* (IOS 5.2–8.2 mm, INS 2.7–4.7 mm N=20), *R. jiemuxiensis* (IOS 4.3–7.5 mm, INS 2.5–3.8 mm); internarial distances larger than interorbital distances of in *R. maoershanensis* (IOS 3.1–3.3 mm, INS 4.5–5.5 mm, N = 3)); (5) interorbital distances larger than width of upper eyelid in *R. dabieshanensis* (IOS 3.1–5.5 mm, UE 2.9–3.8 mm N = 8) (vs. interorbital distances almost equal to upper eyelid in *R. hanluensis* (IOS 3.3–4.5 mm, UE 3.1–4.3 mm N = 16), *R. huanrenensis* (IOS 2.9–4.0 mm, UE 3.0–4.0 mm N = 15) and *R. chensinensis* (IOS 2.9–4.0 mm, UE 3.0–4.0 mm N = 8); upper eyelid interorbital larger to interorbital distances in *R. zhenhaiensis* (IOS 2.3–3.4 mm, UE 3.5–4.5 mm N = 25), *R. kukunoris* (IOS 2.4–3.1 mm, UE 3.8–4.9 mm N = 5), *R. dybowskii* (IOS 3.0–4.0 mm, UE 4.1–5.8 mm N = 25) and *R. amurensis* (IOS 2.6–3.5 mm, UE 3.5–5.2 mm N = 21)); (6) distinct canthus rostralis (vs. not distinct canthus rostralis in *R. longicrus*); (7) the relative finger lengths IV > I > III > II in *Rana dabieshanensis* sp. n (vs. finger lengths III > IV> I > II of *R. hanluica*, *R. luanchuanensis*, and *R. longicrus*); (8) thicker lower arm, LAD 4.9–8.1 mm in males (LAD/SVL radio 0.13) and 5.1–5.3 mm in females (LAD/SVL radio 0.10) (vs. LAD 3.6–4.5 mm (LAD/SVL 0.09) in males (N = 8) and 3.4–4.5 mm (LAD/SVL 0.08) in females (N = 7) of *R. chensinensis*). (9) long hind limb (HLL 100.4–129.1 mm N = 8) (vs. in *R. chaochiaoensis* (HLL 92.0–100.0 mm N = 20), *R. longicrus* (HLL 70.8–84.8 mm N = 20), *R. zhenhaiensis* (HLL 73.4–100.0 mm N = 25), *R. chensinensis* (HLL 80.0–97.0 mm N = 8), *R. kukunoris* (HLL 80.0–99.0 mm N = 5), *R. arvalis* (HLL 61.1–82.4 mm N = 16) and *R. huanrenensis* (HLL 61.4–84.5 mm N = 15); (10) toes being webbed on two thirds (vs. toes fully webbed in *R. chaochiaoensis* and *R. huanrenensis*); (11) larger body sizes, SVL: males 50.9–62.8 mm, N = 8 and females 53.0–68.3 mm, N = 2 (vs. SVL: males 35.6–49.9 mm and females 34.1–53.6 mm in *R. jiemuxiensis*, SVL: males 39.0–46.9 mm, N = 15 and females 42.4–49.0 mm, N = 8 in *R. huanrenensis*, SVL: males 27.2–33.0 mm, N = 12 and females 23.7–41.2 mm, N = 25 in *R. luanchuanensis*); (12) a straight distinct dorsolateral fold lasting from the temporal region to groin in *Rana dabieshanensis* sp. n (vs. dorsolateral fold curved above the tympanum of *R. longicrus*, *R. zhenhaiensis*, *R. maoershanensis*, *R. jiemuxiensis*, *R. chensinensis*, *R. huanrenensis*, *R. arvalis*, *R. amurensis*, *R. asiatica*, *R. dybowskii*, *R. kukunoris*, *R. kunyuensisR. luanchuanensis*, and *R. culaiensis*).

## Discussion

The Chinese species of the genus *Rana* were divided into three species groups based on external morphology (Fei et al. 2009); however, previous studies have been hampered by sampling restricted geographic regions or limited species groups with limited gene markers, mostly based on mtDNA. For example, the European species *R. arvalis* and Central Asian species *R. asiatica* once were considered belong to *R. chensinensis* group (Fei et al. 2009), while phylogenetic analyses indicated that these two species belonged to the same clade with *R. temporaria*. The recent progress on multilocus phylogeny of the genus *Rana* (Yuan et al. 2017, Zhao et al. 2017) indicated seven groups within the Chinese *Rana* *sensu lato* which contained 24 species ( 2017, Zhao et al. 2017). Species in the *R. longicrus* group mostly occur in the southern part of China and Taiwan with no significant changes, except that the number of species in this group has increased. The species of the *R. longicrus* group appear to have highly conserved morphological characteristics compared to other species groups of Chinese *Rana*, implying that this group contains many cryptic species (Yan et al. 2011). In recent years, many species were identified based on molecular identification methods, providing a new understanding of the taxa that were once misidentified. For example, *R. jiemuxiensis* was distinguished from *R. hanluica* based on results of molecular analyses and differing breeding habits (Yan et al. 2011).

In the last decades, different opinions have been proposed on the distribution of *Rana* species in the Dabie Mountains of central China. Initially, the brown frog species found in the Dabie Mountains was identified as *R. japonica* Boulenger, 1879 (Zhao and Wu 1974). Subsequently, *R. japonica* was often mentioned in reports and surveys on amphibian fauna of this area (Lu et al. 1999, Shi et al. 2011, Zhao and Wu 1974, Zhang et al. 2000). However, later it was shown that *R. japonica* is only distributed in Japan, while Chinese populations belong to a different species (Maeda and Matsui 1989). In 2011, the *R. zhenhaiensis* was recorded in the Jintangtai National Nature Reserve (Shi et al. 2011) and the Huangbaishan National Forest Park (Wang et al. 2011) during the monitoring of amphibians in the Dabie Mountains. Subsequently, *R. zhenhaiensis* was identified as *R. culaiensis* in Huangbaishan National Forest Park through phylogenetic analyses (Zhao et al. 2015). In addition, the species of *R. chensinensis* was also mentioned as inhabiting in Dabie Mountains during the monitoring of amphibians (Pan et al. 2014), and phylogeography analysis on *R. chensinensis* had never sampled in this mountains area (Che et al. 2007). So, with the help of phylogenetic analyses, three species (*Rana dabieshanensis* sp. n., *R. culaiensis, and R. chensinensis*) are now known to inhabit the Dabie Mountains (Fang 2011, Zhao et al. 2015, Zhou et al. 2012).

During recent research in the Dabie Mountains, many endemic species like *Moschus anhuiensis* (Su et al. 2000) and *Protobothrops dabieshanensis* (Huang et al. 2012) were discovered, indicating that natural resources and animal diversity of this area are still insufficiently studied. With the addition of *Rana dabieshanensis* sp. n the genus now contains 103 known species and 25 species in China. To date, the new species is only known from a small montane area in Anhui Province of central China. However, its range might include other montane areas of central and southern China, so further surveys are urgently needed for investigation of the current distribution and population status of this species.

## Supplementary Material

XML Treatment for Rana dabieshanensis
